# The effects of spaceflight countermeasures on trabecular bone score (TBS) of the lumbar spine

**DOI:** 10.1007/s11657-025-01624-2

**Published:** 2026-01-13

**Authors:** K. D. Anderson, E. R. Spector, R. Ploutz-Snyder, G. Yardley, N. B. Watts, D. Hans, J. D. Sibonga

**Affiliations:** 1 Emory Sports Performance and Research Center (SPARC), Flowery Branch, GA USA; 2 Emory Sports Medicine Center, Atlanta, GA USA; 3 Emory University School of Medicine, Atlanta, GA USA; 4 KBR, Houston, TX USA; 5 University of Michigan, Ann Arbor, MI USA; 6 Mercy Health, Cincinnati, OH USA; 7 Lausanne University and Hospital, Lausanne, VD Switzerland; 8 NASA Johnson Space Center, SK/311, NASA Parkway 2101, Houston, TX 77058 USA

**Keywords:** Long duration spaceflight, Bone, DXA, BMD, Microarchitecture

## Abstract

**Summary:**

This study evaluated lumbar spine bone mineral density and trabecular bone score in ISS astronauts using DXA under 3 different countermeasure regimes. ARED + bisphosphonate maintained BMD, while both ARED and ARED + bisphosphonate preserved TBS. TBS supplements DXA assessments of spaceflight effects on bone health.

**Purpose:**

To assess the utility of lumbar spine (LS) bone mineral density (BMD) and trabecular bone score (TBS) from scans using dual x-ray absorptiometry (DXA) performed in astronauts before and after spaceflights aboard the International Space Station (ISS). The influence of mission duration and of inflight countermeasures on changes described by BMD and TBS was also evaluated from longitudinal DXA tests performed postflight.

**Methods:**

Preflight and postflight DXA scans, from which BMD and TBS were acquired, were performed from 51 crewmembers: 41 men (mean age ± SD, 48 ± 5, range 37–56 years) and 10 women (mean age ± SD, 44 ± 3 years, range 41–50 years) who flew on missions of ~ 6 months duration. Participants were categorized into three groups: Pre-ARED (advanced resistive exercise device) (*n* = 24), ARED (*n* = 20), and ARED + bisphosphonate (alendronate 70 mg/week) (*n* = 7). Longitudinal DXA scans were obtained from 311 individual astronauts (266 men, 45 women; age range, 41–80 years) from November 1999 to February 2014 to assess trends following spaceflights, categorized as short-duration (less than 1 bone remodeling cycle [BRC]) and long-duration (> 1 BRC).

**Results:**

Only the ARED + bisphosphonate group was not different from preflight BMD (+ 2.8%, *p* = 0.1). Regarding TBS, both the ARED and ARED + bisphosphonate countermeasure groups were not different from preflight (+ 0.2%, *p* = 0.7; −1.5%, *p* = 0.3). Longitudinal trends of TBS and BMD from short-duration missions revealed declining trends in men. In terms of long-duration missions, there tended to be declining trends in spine BMD and TBS when plotted as a function of age.

**Conclusion:**

DXA can detect how various in-flight countermeasures and the length of mission affect the lumbar spine that is enhanced with the addition of TBS.

## Introduction

Since the inception of long-duration missions to the International Space Station (ISS) around 2000, the impact of long-duration spaceflight on the skeletal system has been an ongoing concern. By NASA definition, the distinction of long-duration spaceflight is a mission greater than 30 days, but more recently, long-duration refers to routine ISS missions of 6 months. With multiple missions beyond 6 months planned before the decommissioning of the ISS in 2030 and longer stays on the Moon and Mars projected as part of the Artemis program, safeguarding astronaut skeletal health and evaluating the efficacy of countermeasures have become even more important. Preflight and postflight dual-energy x-ray absorptiometry (DXA) measurements of BMD are required for all long-duration astronauts [1] to describe the effects of prolonged space exposures, the mitigation of effects by spaceflight countermeasures, and the recovery to preflight status after return to Earth. DXA is also performed triennially in all active astronauts (since 1997) and retired astronauts (since 2006) [2] to assess how spaceflight-induced changes might increase the risk for premature osteoporosis in the astronaut after return to Earth.

The skeletal health of astronauts is monitored at NASA Johnson Space Center (JSC) by DXA scans at skeletal sites prone to age-related fragility fractures such as the lumbar spine and proximal femur. DXA is a measurement of areal bone mineral density (BMD, g/cm^2^) that has significant clinical use in the diagnosis of osteoporosis. However, it is important to note that DXA effectively averages bone mass in cortical and trabecular compartments (grams/cm^2^ of the projected bone area) and therefore cannot discern specific effects on cortical bone vs. trabecular bone of the lumbar spine [3, 4]. As a result, the NASA standard that dictates that astronauts must have a BMD derived *T*-score for the lumbar spine that is no less than −1.0 at the time of launch (NASA Std. 3001) [1, 5] could be enhanced by a measurement that can specifically assess trabecular microarchitecture.

The Bone Discipline Team at NASA JSC is particularly concerned that astronauts serving on long-duration missions may be at risk for disruptions in trabecular microarchitecture and premature fragility fractures of the lumbar spine. In previous studies, Russian cosmonauts and US crewmembers on board the ISS lost BMD of the lumbar spine at the rate of ~ 1% per month [6], which is markedly accelerated (up to 10–15×) compared with rates of age-related loss on Earth [7]. The accelerated decline in LS BMD (1–1.5% BMD per year) [7] is associated with disrupted trabecular microarchitecture in women after menopause [8], suggesting that the more rapid bone loss observed in astronauts could similarly disrupt trabecular microarchitecture and increase the risk for vertebral compression fractures in the lumbar spine [9]. Recent data also indicate a delayed or incomplete recovery in trabecular bone of the hip by 2 years after return to Earth (as determined by quantitative computed tomography), in spite of restored BMD of total hip [10]. This lack of recovery after 2 years suggests that irreversible changes to trabecular microarchitecture might have occurred. Additional evidence suggests that spaceflight may cause changes in the trabecular compartments of the hip and lumbar spine that may not recover during postflight rehabilitation [10–12]. However, non-invasive tests for quantifying parameters of trabecular microarchitecture in these skeletal regions or for assessing the efficacy of mitigating countermeasures are only now emerging. This technology gap is especially critical for the vertebrae because of their high trabecular bone content and possible increased risk for vertebral compression fractures due to irreversible losses in trabecular connectivity.

Trabecular bone score (TBS) is a clinically available, easily performed, DXA-derived estimate of trabecular microarchitecture that could be a useful addition to astronaut skeletal health assessments. TBS is a gray-level textural analysis of the 2D lumbar spine DXA images that can be used to extrapolate information reflecting the organization of 3D trabecular microarchitecture. The strong correlation of TBS with parameters of trabecular microarchitecture (e.g., structural model index, trabecular thickness, trabecular separation, trabecular number), measured through 2D histomorphometry and 3D computed tomography [13], suggests that TBS would capture the impact of spaceflight-induced bone loss on the integrity of trabecular bone microarchitecture.

In this study, we first determined the effects of 3 different exercise regimes defined in relation to the use of the advanced resistive exercise device (ARED): Pre-ARED, ARED, and ARED + bisphosphonate on LS BMD and TBS of the lumbar spine in astronauts who flew on ~ 6-month missions on board the ISS. We then constructed curvilinear models of LS BMD and TBS data from male and female astronauts as a function of age and either short or long spaceflight durations. The primary hypothesis of this study was that TBS would detect spaceflight changes to bone that LS BMD alone could not detect and that the combination of resistive exercise with a bisphosphonate drug as a biochemical countermeasure would maintain more astronauts at their preflight skeletal status than with resistive exercise alone [3]. Secondarily, we hypothesized that, because cortical bone is approximately 4× the density of trabecular bone, LS BMD and TBS data would demonstrate independent trends during the postflight period because TBS would not be masked by dense cortical bone. Results from this study will determine the utility of TBS as an additional metric in evaluating the effectiveness of spaceflight countermeasures and potential long-term consequences of spaceflight on the skeleton.

## Methods

### Study subjects

Preflight and postflight DXA was obtained from 51 crewmembers: 41 men (mean age ± SD, 48 ± 5, range 37–56 years) and 10 perimenopausal women (mean age ± SD, 44 ± 3 years, range 41–50, years) who flew on missions of ~ 6 months duration (group mean duration 5.4 months, Table 1). No astronaut subjects reported a history of chronic kidney disease, diabetes, or chronic glucocorticoid use prior to the mission. The preflight DXA was obtained within 1 year of spaceflight while the postflight DXA was obtained within 1 month of return to Earth. Participants were categorized into three groups based on access to the advanced resistive exercise device (ARED), an ISS exercise hardware that simulates weight-lifting (as much as 600 lb-ft [14]) in the microgravity of space with or without the intake of an anti-resorptive therapy. In this report, these groups are identified as: Pre-ARED astronauts (*n* = 24) whose spaceflights pre-date ARED availability on ISS (2000–2009); ARED astronauts (*n* = 20) with access to ARED during Spaceflight (2009–present); and [3] ARED + bisphosphonate (ARED + BP) astronauts (*n* = 7) with access to ARED combined with a weekly dose of the bisphosphonate alendronate (70 mg/week) initiated 3 weeks prior to launch and continued throughout the mission [3, 15, 16]. Astronauts were adherent to their exercise countermeasure group throughout spaceflight. Upon return to Earth, astronauts discontinued use of the ARED and bisphosphonate medication. The efficacy of resistive exercise performed on the ARED to mitigate DXA-measured BMD of the lumbar spine, combined with or without the bisphosphonate countermeasure, has been reported previously [3, 16].

**Table 1:** Astronaut age and flight duration by group (mean ± standard deviation). By single-factor analysis of variance, differences were not significant (*p* = 0.14 for age and *p* = 0.17 for flight duration)

Group	Age (years)	Flight duration (days)
Pre-ARED *n* = 24 (20 men, 4 women)	46.2 ± 4.5	172 ± 28
ARED *n* = 20 (15 men, 5 women)	48.1 ± 4.3	157 ± 28
ARED + BP *n* = 7 (6 men, 1 woman)	49.5 ± 4.1	164 ± 15

For crew skeletal health and risk surveillance performed NASA JSC, triennial DXA scans are performed in all active astronauts (required medical testing) and offered to retired astronauts (for long-term health surveillance) [2]. Longitudinal DXA measurements collected between November 1999 and February 2014 from 311 individual astronauts (266 men, 45 women; age range, 41–80 years) were analyzed. Trends in LS BMD and TBS of short duration or long duration of men or women were plotted as a function of the astronaut`s age at DXA testing, regardless of countermeasure strategy. Based upon an approximate duration for a bone remodeling cycle (BRC) of at least 100 days [17], spaceflight exposures were categorized as short-duration (< 1 BRC, i.e., 0–99 days) or long-duration (≥ 1 BRC, ≥ 100 days). DXA observations categorized as short duration were composed of measurements conducted in astronauts who have flown in space but never flown on a long duration spaceflight, while DXA data denoted as long duration were all acquired in astronauts after flying their first long duration mission. Measurements from DXA scans performed within the first 3 years after a long duration astronaut returned to Earth were excluded from plots to displaying postflight trends. This exclusion was based upon our previous assertion that BMD deficits in long duration astronauts following spaceflight were substantially restored by 3 years after return to Earth [18].

### DXA acquisition

DXA images obtained preflight and postflight [1, 19] for an astronaut on a single spaceflight were acquired by one DXA operator using the same scanner and software (Discovery W, Hologic, Waltham, MA) at NASA JSC, Houston TX while the longitudinal DXA images from either the triennial testing of active astronauts or retired astronauts were performed by multiple DXA operators (*n* = 5) across ~ 20 years. During the period of data acquisition, the coefficient of variation (CV) for all operators was less than 2% for BMD measurements and 5.4% for TBS measurements of the lumbar spine.

**Fig. 1:**
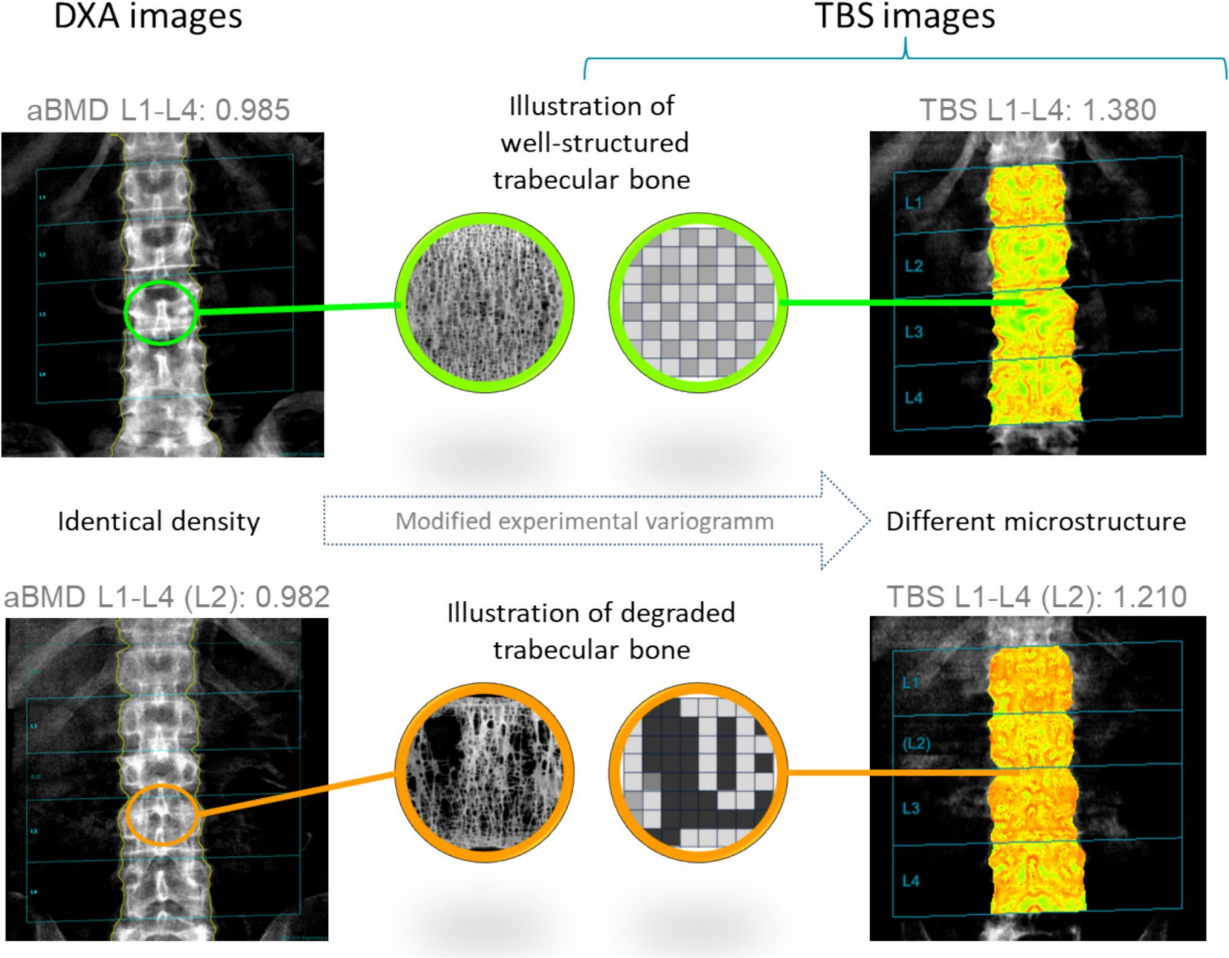
Illustration of the TBS principle: 2D projection images of the lumbar spine from two subjects having similar density but different underlying microarchitecture and illustrated by different TBS values. A high TBS value is compatible with a well-structured bone, and a low TBS value with a degraded trabecular bone poorly connected (images courtesy of Medimaps Group USA, LLC)

Areal BMDs and TBS were measured at the lumbar spine (L1–L4). TBS was acquired using the TBS iNsight 2.1.0 software (Medimaps group, Geneva, Switzerland) which captured 3D texture information from gray-level DXA-derived images. Pixel intensities were mapped on a 2D projection image, and field function changes were estimated through a modified and proprietary experimental variogram [20]. A representative graphical depiction of the pattern generated for TBS is shown in Fig. 1 (courtesy of Medimaps group).

### Data analysis

Astronaut age and flight duration for the preflight to postflight comparison were first analyzed by group (Pre-ARED, ARED, and ARED + BP) using a one-way ANOVA. Statistical significance was considered *p* < 0.05. Group mean ± SD values for astronaut age and flight duration were calculated and presented in Table 1. Preflight and postflight DXA scans were then anonymized and exported to a study collaborator (N. Watts) who generated TBS values (Mercy Health, Cincinnati, OH). The resulting data were then analyzed at JSC using a two-tailed, paired Student’s *t*-test to detect differences between preflight and postflight means of the absolute change. Total difference and percent change per month are reported, and statistical significance was considered at *p* < 0.05 and presented in Table 2. The measurement error (least significant change, LSC) for BMD and TBS measurements was calculated using the equation LSC = (CV * 2.77) * (BMD or TBS mean) where, as previously mentioned, the CV for BMD was 2% and the CV for TBS was 5.4% consistent with International Osteoporosis Foundation (IOF) standards [21]. The resulting LSC was then used as a threshold to determine the number of individual astronauts that had biologically significant preflight to postflight changes from a 6-month mission duration.

**Table 2:** LS BMD and TBS by group (mean ± SD, with ranges in parentheses). Since not all mission durations were equal, changes are reported as both absolute change from preflight to postflight and as percent change per month of flight. *p < 0.01, **p = 0.01

Countermeasures group	Pre-ARED (*n* = 24)	ARED (*n* = 20)	ARED + BP (*n* = 7)
Preflight BMD (g/cm^2^)	1.070 ± 0.10 (0.901 to 1.224)	1.092 ± 0.11 (0.918 to 1.281)	1.040 ± 0.14 (0.920 to 1.329)
Postflight BMD (g/cm^2^)	1.023 ± 0.09 (0.878 to 1.176)	1.071 ± 0.11 (0.855 to 1.242)	1.067 ± 0.14 (0.939 to 1.350)
BMD absolute change (g/cm^2^)	−0.047 ± 0.03* (−0.13 to −0.0003)	−0.021 ± 0.03* (−0.10 to 0.03)	0.028 ± 0.04** (−0.13 to 0.10
BMD %change/mo	−0.75 ± 0.49 (−1.9 to −0.07)	−0.35 ± 0.54 (−1.5 to 0.59)	0.57 ± 0.86 (−0.22 to 0.10)
Number exceeding BMD negative LSC	15	9	0
Preflight TBS (g/cm^2^)	1.441 ± 0.07 (1.301 to 1.569)	1.420 ± 0.08 (1.246 to 1.539)	1.422 ± 0.07 (1.339 to 1.541)
Postflight TBS (g/cm^2^)	1.404 ± 0.09 (1.206 to 1.530)	1.416 ± 0.08 (1.253 to 1.563)	1.401 ± 0.09 1.338 to 1.589)
Absolute (%change)	−0.043 ± 0.04 * (−0.13 to 0.04)	0.003 ± 0.03 (−0.08 to 0.07)	−0.022 ± 0.05 (−0.08 to 0.05)
TBS %change/mo	−0.53 ± 0.54 (−1.7 to 0.41)	0.03 ± 0.50 (−1.2 to 1.0)	−0.28 ± 0.67 (−1.0 to 0.57)
Number exceeding TBS negative LSC	11	2	3

The longitudinal DXA scans were analyzed at JSC with TBS iNsight® software v2.1.0 (Medimaps group, Geneva, Switzerland) installed on the JSC computer that is used to assess LS BMD from DXA scans. Nonlinear models of the 1175 scans data were plotted relative to astronaut age to describe trends in LS BMD and TBS. Trends for men and women were assessed separately with regard to exposures to either short-duration or long-duration spaceflight. The presence of co-variates, e.g., chronic kidney disease, chronic glucocorticoid therapy, in astronaut subjects did not lead to exclusion of data.

## Results

### Astronaut age and flight duration for preflight to postflight comparison

Astronaut age (Table 1) was not significantly different between Pre-ARED (46.2 ± 4.5 years), ARED (48.1 ± 4.3 years), and ARED + BP (49.5 ± 4.1 years) groups (*p* = 0.14). Astronaut flight duration (Table 1) was also not significantly different between Pre-ARED (172 ± 28 days), ARED (157 ± 28 days), and ARED + bisphosphonate (164 ± 15 days) groups (*p* = 0.17).

### LS BMD and TBS for preflight to postflight comparison

Postflight LS BMD was significantly lower than preflight LS BMD in the pre-ARED group (−4.4%, *p* < 0.001) and ARED group (−1.9%, *p* < 0.01), but not in the ARED + BP group (+ 2.8%, *p* = 0.1) (Table 2). For TBS (Table 2), a decline from preflight was statistically significant in the Pre-ARED group (−3.0%, *p* < 0.01) but was not statistically different from preflight in both the ARED group (+ 0.2%, *p* = 0.7) and the ARED + BP group (−1.5%, *p* = 0.3). For all groups, both total absolute change and percent change per month were highly variable, with the percent change from preflight exceeding the least significant change (LSC). There were 26 astronauts who were significantly below the BMD LSC while there were 3 astronauts who were below the TBS LSC. The number of subjects that were below the BMD LSC and TBS LSC according to countermeasure group is presented in Table 2.

### Curvilinear trends of short duration and long duration astronauts

**Fig. 2:**
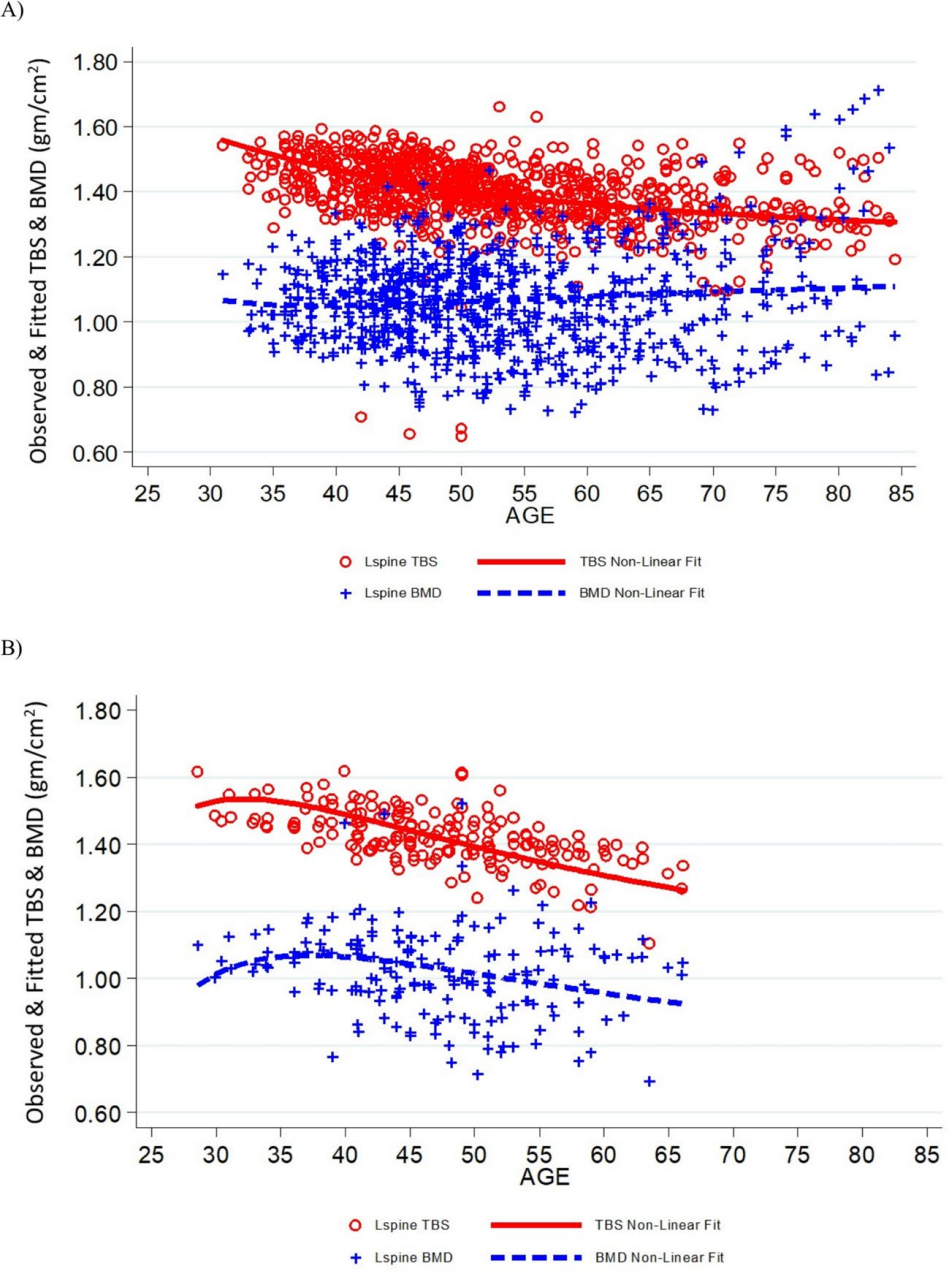
Curvilinear fit of TBS and BMD data of male astronauts following short-duration missions **A** and long-duration missions **B**. Data are plotted by age at the time of DXA measurement to show temporal changes in the cohort during recovery and afterward. Blue plus symbols represent BMD; open circles represent TBS

**Fig. 3:**
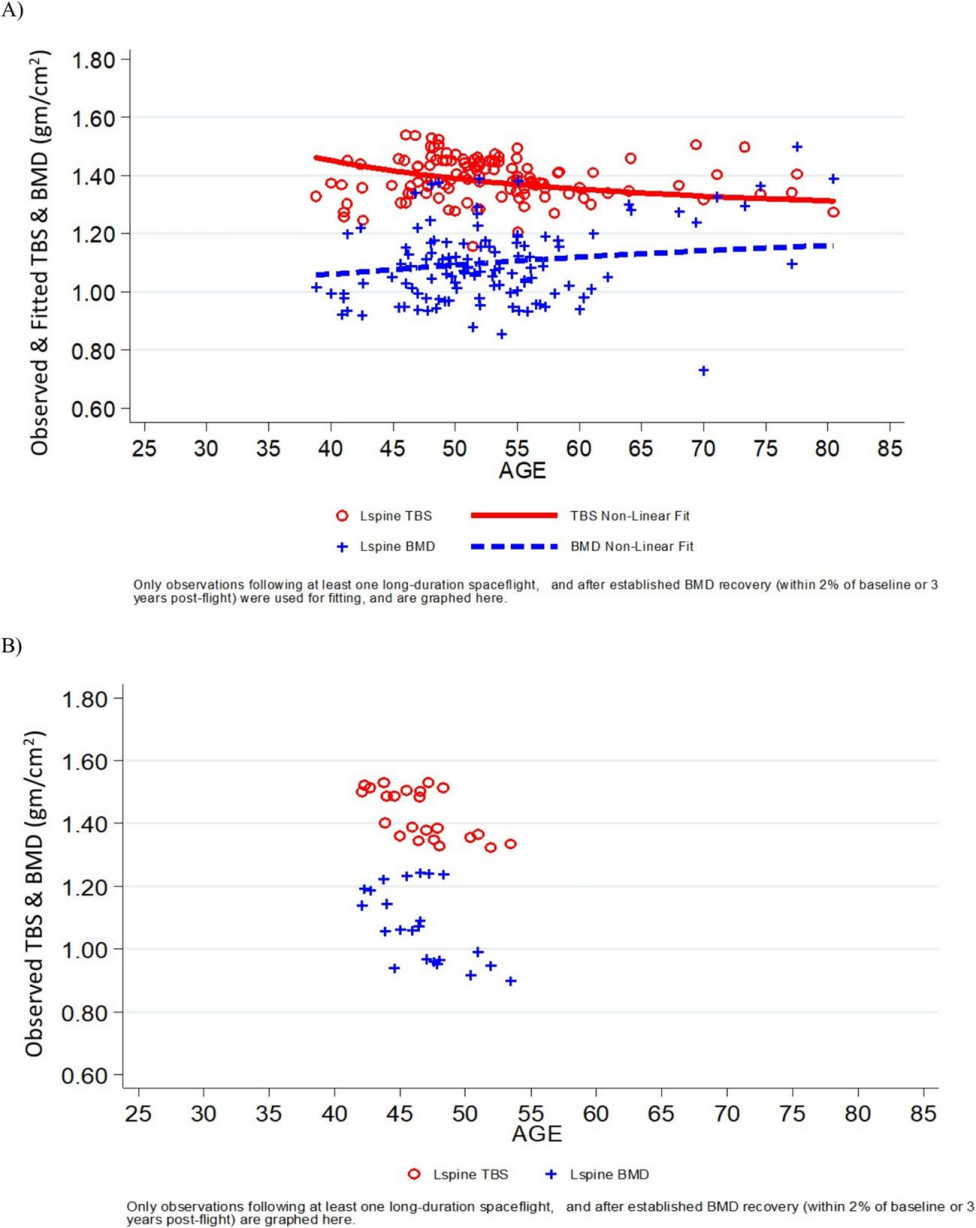
Curvilinear fit of BMD and TBS data from female astronauts following short-duration missions **A** and long-duration missions **B**. Fitted data graphed here are for DXA measurements conducted following at least one long-duration spaceflight, and after established BMD recovery (i.e., within 2% of preflight measurements) or within 3 years of return to Earth [17]. Blue plus symbols represent BMD; open circles represent TBS

The curvilinear trend of LS BMD of male short-duration astronauts (Fig. 2) increased with age while TBS decreased. Data collection of LS BMD and TBS from male astronauts will decline at advanced ages which could influence the fit of trend lines for long-duration missions. The curvilinear trend of LS BMD of female short-duration astronauts (Fig. 3) also increased with age while TBS decreased. Minimal data were available from female astronauts after long-duration missions to discern any trends (data not shown). Although DXA scans are offered, on a triennial basis, to retirees and astronauts who leave NASA, attrition due to scheduling conflicts, health concerns, or travel difficulties accounts for the reduced data acquisition at advanced ages.

## Discussion

This is the first report of TBS to evaluate the effects of spaceflight on the integrity of trabecular bone in the lumbar spine of astronauts. Our primary hypothesis that TBS would detect effects of spaceflight that LS BMD alone could not detect was supported. TBS detected a beneficial effect of ARED to preserve trabecular integrity while a beneficial effect on LS BMD was not evident. Our results also reflected a significant change in TBS of the lumbar spine in astronauts during prolonged spaceflight exposure and the ability of in-flight countermeasure (ARED alone or in combination with bisphosphonate) to prevent those changes as compared to preflight. Furthermore, LS BMD and TBS of male short and long-duration astronauts demonstrated declines with advancing age. Hence, we consider TBS to be useful in determining the effect of spaceflight countermeasures on trabecular microarchitecture of the lumbar spine and can provide important longitudinal information about astronaut skeletal health additional to that of LS BMD.

We found a significant decrease in postflight DXA-measured BMD of the lumbar spine as compared to preflight in the Pre-ARED and ARED groups. The averaged monthly loss rate of LS BMD among ARED users in this analysis (−0.35%, *n* = 20) was comparable to the average monthly LS BMD loss (0.43%, *n* = 7) reported when the robust exercise hardware (ARED) was first available for limited in-flight use on the ISS [1]. However, a more recent survey of LS BMD data in ISS astronauts who flew between 2009 and 2021, when all crew had access to the ARED (average duration 179 ± 43 days, *n* = 64 total), revealed that 27% of astronauts had at least a 5% decline in LS BMD, more than −0.85% per month (unpublished data). Collectively, these data raise a concern that, despite exercise on the ARED, a risk for disrupted trabecular microarchitecture remains.

Significant declines in TBS during spaceflight occurred in pre-ARED astronauts. These data are akin to the postflight BMD data (multiple sites) from previous studies that indicated that low-resistance exercise (maximum 300 lb-ft, Pre-ARED) in space does not provide a sufficient stimulus to mitigate skeletal adaptation to reduced weight-bearing function—characterized by stimulated bone resorption and reduced bone mass [3, 15, 16]. However, after the availability of ARED on the ISS, TBS did not show significant change from preflight, whereas BMD of the lumbar spine showed a statistically significant albeit attenuated decline (i.e., ~ 50% BMD relative to the Pre-ARED group). Previous reports have shown that resistance exercise on the ARED (at a greater magnitude of lb-ft) can attenuate the postflight deficit in BMD of the lumbar spine, although elevated levels of bone resorption biomarkers persist [16, 22]. The use of ARED during spaceflight could, for example, be increasing bone formation on the periosteal surface of the vertebrae with no influence on the resorbed surfaces of trabecular bone or of the endocortical surface. This modeling of whole bones was previously seen with QCT scans quantifying radial growth of long bones of crewmembers during the first year of ambulation after return to Earth [9].

Neither LS BMD nor TBS detected significant changes from preflight measurements in the ARED + BP group. We would expect the anti-resorptive actions of the bisphosphonate to mitigate loss in bone mass and maintain microstructure in trabecular bone (i.e., skeletal surfaces in bone marrow easily accessible to osteoclasts [23] as well as reduce porosity and thinning of the cortex. However, we interestingly also found no difference from preflight in TBS in the ARED group alone when trabecular bone loss is well described using similarly sized datasets but measured with quantitative computed tomography [24]. This finding highlights the limitations of TBS, a measure derived from DXA, to detect any disorganization of microarchitecture expected to accompany accelerated trabecular bone loss.

DXA averages cortical and trabecular compartments (grams/cm^2^ of the projected bone area) and cannot separate specific effects on trabecular and cortical compartments [23]. This limitation raises the concern that DXA alone does not give a complete picture of fracture risk [24]. If deficits induced during spaceflight were allowed to persist postflight in an unmitigated or unrestored state, the astronaut may be at increased fracture risk after return to Earth’s 1G with the resumption of typical preflight activities. Unrestored deficits might also combine with the effects of normal aging to put the astronaut at earlier or greater risk for osteoporotic fractures later in life [23, 24]. Even though all 51 ISS astronauts returned from a ~ 6-month ISS mission with a TBS value greater than 1.31 (which is classified as “low risk” for vertebral fracture in the terrestrial aging population), there were 26 astronauts that exceeded the BMD least significant change (LSC) and 3 astronauts that also exceeded the TBS LSC. Exceeding the BMD LSC indicates there was a statistically meaningful loss in BMD during spaceflight, and exceeding the TBS LSC warrants closer monitoring of long-term skeletal health in those individuals. To determine the potential longitudinal consequences of premature osteoporosis, we also performed TBS analysis of the triennial longitudinal DXA scans of retired astronauts.

The short-duration curvilinear plots (Figures 2, 3) displayed similar trends for TBS and LS BMD in observations conducted in male and female astronauts. This observation is perhaps not unexpected, given that short-duration spaceflight is typically only a few weeks in duration. This exposure to spaceflight would therefore not be long enough to influence one complete cycle of bone remodeling [17]. These curvilinear trends showed general declines in both TBS and LS BMD relative to astronaut age at the time of DXA measurements. Menopause-induced estrogen deficiency is a widely recognized risk factor for skeletal fragility in women; it would be important to monitor changes in female astronauts to intervene (if needed) at an appropriate time (before, during, or after spaceflight) to prevent the combination of bone loss due to spaceflight and aging.

As mentioned, long-duration model data from male astronauts (Fig. 2A) showed that the BMD data for the lumbar spine appears to scatter upward at advanced ages, likely due to degenerative changes, while TBS continues to decline. There were too few long-duration female astronauts (*n* = 8) to draw any sex-specific conclusions (Fig. 3B). TBS could enhance the postflight DXA surveillance of skeletal changes by concurrently assessing trabecular disorganization. Complete recovery of LS BMD could occur within one year of re-adaptation to the terrestrial environment (*n* = 8 astronauts) [25]. This projection is supported by a recovery trend modeled with data from a larger study in astronauts (*n* = 45), which further suggests that 50% of LS BMD losses occur during the 5 months after their return to Earth [6]. The decline in TBS after return to Earth reported herein suggests that long-duration spaceflights may compromise trabecular microstructure, despite continual recovery of bone mineral density [25]. Our data further supports previous findings that postflight bone strength remains reduced after complete restoration of BMD [26].

Disrupted trabecular microarchitecture is an established risk factor for vertebral fractures [8]. TBS measurements of trabecular bone have been associated with histomorphometric changes of iliac crest biopsies in young patients with idiopathic osteoporosis [13]. TBS has also been used to detect disorganized trabecular bone with estrogen deficiency [27] and breast cancer in menopausal women [28] and shows the long-term treatment response to the bisphosphonate zoledronic acid [29], as well as stem cell transplant [30]. Additionally, low TBS is associated with increased fracture risk independent of LS BMD and is a risk factor which could provide a complementary bone assessment tool to evaluate the bone health status of astronauts [13, 31, 32]. Finally, TBS analysis can be easily done on a previously performed DXA imaging without the need for re-imaging and additional exposure to ionizing radiation, which is a major concern for astronauts. The assessment of both BMD and TBS from LS DXA scans would enhance the overall evaluation of skeletal health after spaceflight and characterization of skeletal countermeasures being tested in-flight.

In addition, our findings from the ISS agree with the literature suggesting that TBS and LS BMD detect independent effects of countermeasures [4] which have been tested in-flight. We previously observed that astronauts who recover BMD in the proximal femur still had compromised bone strength as indicated by the analysis of finite element models [33]. Thus, it may be beneficial for future work to correlate TBS with vertebral bone strength (e.g., estimated from finite element models) [34] for these trabecular-rich bones. DXA scanning is required to be performed preflight and postflight in long-duration astronauts. Thus, TBS analysis would be done on those same DXA scans incurring no additional exposure to ionizing radiation or increased time beyond required crew testing schedules.

This study has several limitations. Firstly, the number of long-duration female astronauts was small (8:1 men to women), making it challenging to draw any sex-specific conclusions. All available data from female astronauts were included regardless of age, which means we did not account for the potential confounding effects of hormonal changes associated with contraceptive use, perimenopause, or menopause. This lack of consideration of age-related hormonal changes could influence the outcomes and interpretations related to long-term bone health in women. Additionally, the findings may have been affected by the insufficient duration of the spaceflight countermeasures, so additional studies should consider longer durations to fully understand their impacts. Furthermore, with even longer duration missions, there will be an opportunity to investigate and describe the effects of extended spaceflight on trabecular bone score (TBS) more comprehensively. Future research could provide valuable insights into the long-term impacts of spaceflight on bone health, potentially leading to more effective countermeasures for preserving astronaut skeletal integrity during prolonged missions.

The purpose of this study was to investigate TBS in the overall bone health assessment among astronauts and to evaluate whether it is complementarity to LS BMD that adds to our understanding of the specific bone health changes seen in this population. A recent analysis of postflight fractures in the astronaut corps determined an increased rate of fractures in the spine (and hip) of astronauts previously exposed to a long-duration spaceflight mission (i.e., a spaceflight duration of months vs. weeks) (submitted manuscript under review). Based on our current findings, we recommend integrating TBS into normal monitoring procedures at NASA Johnson Space Center as a method of acquiring additional information on the bone health of astronauts without additional radiation exposure. This additional measure will be a valuable resource to estimate changes in trabecular microstructure, enhance the assessment of fracture risk in astronauts irrespective of the LS BMD *T*-score values, and be used to evaluate new countermeasures for protecting the skeleton during long-duration spaceflight.

## Data Availability

Available upon reasonable request.

## Abbreviations

BMD: Areal bone mineral density
DXA: Dual-energy X-ray absorptiometry
TBS: Trabecular bone score
ARED: Advanced resistive exercise device
NASA: National Aeronautics and Space Administration
JSC: Johnson Space Center
ISS: International Space Station
